# Analysis of Genetic Variation and Phylogeny of the Predatory Bug, *Pilophorus typicus*, in Japan using Mitochondrial Gene Sequences

**DOI:** 10.1673/031.011.0118

**Published:** 2011-02-16

**Authors:** Katsura Ito, Hiroshi Nishikawa, Takuji Shimada, Kohei Ogawa, Yukio Minamiya, Masafumi Tomoda, Kengo Nakahira, Rika Kodama, Tatsuya Fukuda, Ryo Arakawa

**Affiliations:** ^1^ JST Innovation Satellite Kochi; Tosayamada, Kami, Kochi 782–8502, Japan; ^2^ Laboratory of Applied Entomology, Faculty of Agriculture, Kochi University; Monobeotsu, Nankoku, Kochi 783- 8502, Japan; ^3^ Present address: Institute of Biological Control, Faculty of Agriculture, Kyushu University; Higashi-ku, Hakozaki, Fukuoka 812–8581, Japan

**Keywords:** biological control, cytochrome B (*cytB*), cytochrome oxidase subunit I (*COI*), indigenous natural enemy, phylogenetic analysis

## Abstract

*Pilophorus typicus* (Distant) (Heteroptera: Miridae) is a predatory bug occurring in East, Southeast, and South Asia. Because the active stages of *P. typicus* prey on various agricultural pest insects and mites, this species is a candidate insect as an indigenous natural enemy for use in biological control programs. However, the mass releasing of introduced natural enemies into agricultural fields may incur the risk of affecting the genetic integrity of species through hybridization with a local population. To clarify the genetic characteristics of the Japanese populations of *P. typicus* two portions of the mitochondrial DNA, the cytochrome oxidase subunit I (*COI*) (534 bp) and the cytochrome B (*cytB*) (217 bp) genes, were sequenced for 64 individuals collected from 55 localities in a wide range of Japan. Totals of 18 and 10 haplotypes were identified for the *COI* and *cytB* sequences, respectively (25 haplotypes over regions). Phylogenetic analysis using the maximum likelihood method revealed the existence of two genetically distinct groups in *P. typicus* in Japan. These groups were distributed in different geographic ranges: one occurred mainly from the Pacific coastal areas of the Kii Peninsula, the Shikoku Island, and the Ryukyu Islands; whereas the other occurred from the northern Kyushu district to the Kanto and Hokuriku districts of mainland Japan. However, both haplotypes were found in a single locality of the southern coast of the Shikoku Island. *COI* phylogeny incorporating other *Pilophorus* species revealed that these groups were only recently differentiated. Therefore, use of a certain population of *P. typicus* across its distribution range should be done with caution because genetic hybridization may occur.

## Introduction

Introducing natural enemies as control agents for agricultural pests has long been attempted in hope of long lasting suppression effects, reducing pesticide chemicals, saving labor, cutting costs, etc. However, introducing an alien natural enemy into a new agroecosystem may incur ecological and genetic risks. Ecologically, they may secondarily attack non-target insects and drive them into extinction (reviewed in Howarth 1991; Simberloff and Stiling 1996). Genetically, the mass release of introduced natural enemies into agricultural fields may affect the genetic integrity of a local population of species through hybridization. To avoid these risks, utilization of indigenous natural enemies, i.e. mass-reared predators collected from the local area, has been attempted by release into agricultural fields because their ecology and genetic background may be similar to the local population as compared to one that is exotic, and thus may more easily adapt to the local environment with fewer risks. However, geographic proximity does not necessarily reflect genetic distance. For example, a recent phylogenetic study showed that close local populations of a parasitic wasp that were used as a natural enemy was composed of multiple cryptic strains that were different in host use and other life histories (Phillips et al. 2008). Thus, phylogenetic analyses can provide primary data of genetic structure of an indigenous natural enemy, allowing inference about ecological and genetic consequences in the application field.

*Pilophorus typicus* (Distant 1909) (Heteroptera: Miridae) is a candidate as an indigenous natural enemy in biological control programs in Japan. This is polyphagous predatory bug that looks like an ant (Ito et al. 2010). This species occurs in Japan, Taiwan, China, the Philippines, Indochina, Malaysia, Indonesia, Sri Lanka, and India (Schuh 1984). In Japan, this species is distributed from the Ryukyu Islands to Honshu of the mainland (Yasunaga 2001). Adults (approximately 2.7 mm long) and larvae are usually found on various wild plants and greenhouse crops (Yasunaga 2001). Because *P. typicus* preys on various agricultural pests such as whiteflies, thrips, and spider mites (H. Nishikawa et al. unpublished data) that damage commercially important vegetables such as tomato, eggplant, and green pepper under greenhouse conditions. However, degrees of genetic differentiation among geographic populations of *P. typicus* are presently completely unknown.

In various insect groups, nucleotide sequence information of several gene regions on mitochondrial DNA (mtDNA) has been used for evaluating phylogenetic relationships among closely related species or genetically heterogeneous populations of a single species because these regions show sufficiently high rates of nucleotide substitution (e.g. Hebert et al. 2003; Pons et al. 2004; Havill et al. 2007). In particular, the cytochrome oxidase subunit I (*COI*) has been most frequently used in phylogenetic analyses (Hebert et al. 2003), or studies of the genetic structure of agricultural pests (Smith 2005). The cytochrome B (*cytB*) gene has been proved to have the same level of sequence variation as the *COI* region for phylogenetic analysis of many insect orders (Simmons and Weller 2001), and though used less frequently than *COI,* this region has been used for phylogenetic analyses in Heteroptera (e.g. Muraji et al. 2000a, 2000b, 2001). In this study, partial regions of the *COI* and *cytB* genes of *P. typicus* specimens collected from a wide range of Japan were sequenced, and attributes of sequence variation in each region as well as phylogenetic relationship within *P. typicus* using combined sequences were investigated. In addition, the degree of the sequence variation was compared with that found between other *Pilophorus* species to infer the taxonomic status of the phylogenetic groups.

## Materials and Methods

### Mites

Sixty-four individuals of *P. typicus* sampled from 55 localities covering the Ryukyu Islands and the Japanese mainland from Kyushu to Honshu were used for the analysis of the *COI* and *cytB* sequences (Table 1). One individual was analyzed for 47 localities, two for 7 localities, and three for 1 locality. One individual *P. setulosus* collected in the Hokuriku district was sequenced and used as an outgroup. All sample individuals were stored at -30° C until DNA extraction.

### PCR and sequencing procedure

The whole body of a sample individual was ground with a plastic pestle in a 1.5 ml microcentrifuge tube containing 200 μl of HMW buffer (10 mM Tris, 150 mM NaCl, 10 mM ethylenediaminetetraacetic acid (EDTA)2Na (pH 8.0), 1.255% (w/v) sodium dodecylsulfate (SDS) and 0.1 mg/ml proteinase K). After incubation of the mixture at 55° C for 30 min, 500 μl phenol-saturated with TE buffer (10 mM Tris-HCl, 1 mM EDTA, pH = 8) were added and mixed thoroughly. The mixture was centrifuged at 14,000 rpm for 10 min at 4° C to separate phases. The upper aqueous phase was mixed with 500 μl of chloroform:isoamyl alcohol (24:1) mixture and centrifuged. The upper phase was dissolved in 500 μl of 100% ethanol with 20 μl of 3M sodium acetate to precipitate DNA. The precipitate was collected by centrifugation, washed with 120 μl of 70 % ethanol, partially dried under the vacuum, and then resuspended in 30 μl of TE buffer. DNA samples were stored at -20° C until use.

PCR was performed in a 50 μl reaction mixture containing 1.25 μl of DNA sample, 1 X PCR buffer (10mM Tris-HCl buffer (pH 8.3 at 25° C), 50 mM KCl, and 1.5 mM MgCl_2_); 0.16 mM of each dNTP, 0.3 mM of each primer, and 1.25 U of rTaq DNA polymerase (TOYOBO). After incubation at 94° C for 30 sec, DNA was amplified by 45 cycles of incubation at 94° C for 1 min, 48° C for 2 min, and 72° C for 2 min with a final extension at 72° C for 15 min. The *COI* region was amplified using primers previously reported by Folmer et al. (1994): LCO1490 (5'- GGT CAA CAA ATC ATA AAG ATA TTG G -3') and HC02198 (5'- TAA ACT TCA GGG TGA CCA AAA AAT CA -3'). The *cytB* region was amplified using degenerate primers manually designed from the consensus sequence of the partial *cytB* regions of Miridae and related species deposited in DDBJ/EMBL/GenBank DNA databases (EU401991, AY327435, AY327430, AY916050, DQ372123): *cytB*-F1_10623 (5'-ATT AC(A/T) AAT (T/C)TA CT(A/C) TCA GC-3') and cy*tB*-R1_11002 (5'-CAT TCT GGT TG(A/G) ATG TG(G/T) AC-3'). Attached numbers agree with the annealing positions in reference to the mitochondrial genome of *Lygus lineolaris* (EU401991). After amplification, reaction mixtures were subjected to electrophoresis in 1% low-melting-temperature agarose gels (Agarose-L, NipponGene), and DNA bands stained with ethidium bromide were excised and purified with QIAquick Gel Extraction Kit (Qiagen, www.qiagen.com). Sequence analyses were conducted using a BigDye Terminator v3.1 Cycle Sequencing Kit (Applied Biosystems, www.appliedbiosystems.com) and ABI Prism 3100 Genetic Analyzer (Applied Biosystems) according to manufacturer's instructions. Sequence primers were the same as used in PCR reaction.

**Table 1.**
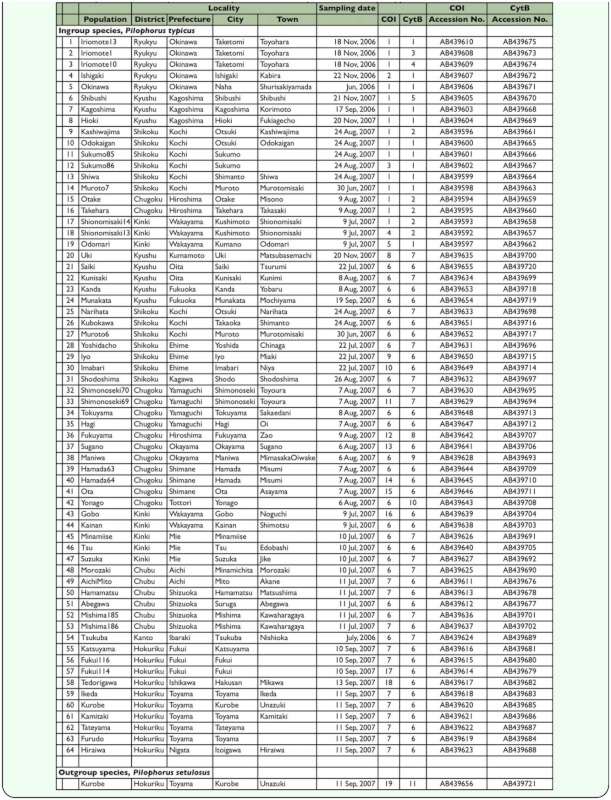
Summary of *Pilophorus* specimens analyzed in this study. Details of haplotypes are summarized in Table 2

Sequence data were aligned using Clustal W 1.83 (Thompson et al. 1994) with default parameter setting. To evaluate data, nucleotide compositions in each codon position, proportions of variable sites, and transition/transversion rates were investigated for each region using MEGA4 software (Tamura et al. 2007). The degree of saturation was assessed for each region by pairwise plotting of the proportion of different sites between two sequences at each codon position against the Tamura-Nei distance (Tamura and Nei 1993) between them including all codon positions. Moreover, genetic divergence within phylogenetic groups was estimated using the number of base substitutions per site from averaging over all sequence pairs within each group (Nei and Kumar 2000). The analyses were conducted using the Tamura-Nei method in MEGA4. To assess the congruence of the two regions, the partition homogeneity test (Farris et al. 1994, 1995) was conducted using the HOMPART command (1000 replicates) implemented on the software PAUP^*^ ver. 4.0b10 (Swofford 2003).

### Phylogenetic analysis of *P. typicus*

As a preliminary test, the phylogenetic analysis based on the neighbor-joining (NJ) method was performed separately for the *COI* and *cytB* regions using MEGA4 to investigate the degree of consistency of mutation patterns in different regions. In these analyses, the nucleotide substitution model for each region was selected using the likelihood ratio test with the program Modeltest 3.7 (Posada and Crandall 1998). Reliability of branches was estimated by 1000 bootstrap resamplings.

The combined sequences were subjected to the phylogenetic analysis of the maximum likelihood (ML) method using the heuristic search algorithm through the HSEARCH command in PAUP^*^. The selection of the nucleotide substitution model and the estimation of the substitution rate matrix were conducted on Modeltest. The starting tree was obtained via the neighbor-joining method, and used for the heuristic search of the ML tree by tree-bisection-reconnection (TBR) swapping (HSEARCH command: criterion = likelihood, addseq = random, nreps = 10). Other parameters were set according to default values in the HSEARCH command. The reliability of internal branches was assessed by 1000 bootstrap resamplings with TBR and the same parameter set as used in constructing the original ML tree.

### Variation within *Pilophorus*

To understand the degree of observed genetic variation in the light of intrageneric variation, the phylogeny of the *COI* region including other *Pilophorus* species was investigated. In addition to three newly obtained sequences of *P. typicus* (Muroto6 and Muroto7) and *P. setulosus,* the sequences of four other species and one unidentified strain of *Pilophorus* (DDBJ/EMBL/GenBank: AY252988, AY253015, AY253025, AY253083, AY253102) were used for the analysis. The tree was rooted with the sequence (EU427341) of an anthocorid bug *Orius niger* (Wolff) (Hemiptera: Anthocoridae), whose life history is similar to *Pilophorus* bugs. ML analysis was conducted using homologous 533 bp as in the above analyses. The *cytB* sequences were not analyzed because of the scarcity of sequence information in *Pilophorus.*

## Results and Discussion

The aligned sequence lengths of the *COI* and *cytB* regions analyzed were 534 and 217 bp, respectively. No insertion or deletion was found in either region. Eighteen haplotypes were detected in the *COI* region, and 10 in the *cytB* region among the 64 individuals of *P.* *typicus* (Table 2). All sequences have been deposited in DDBJ/EMBL/GenBank DNA databases (Accession numbers: AB439592 and AB439721).

**Table 2.**
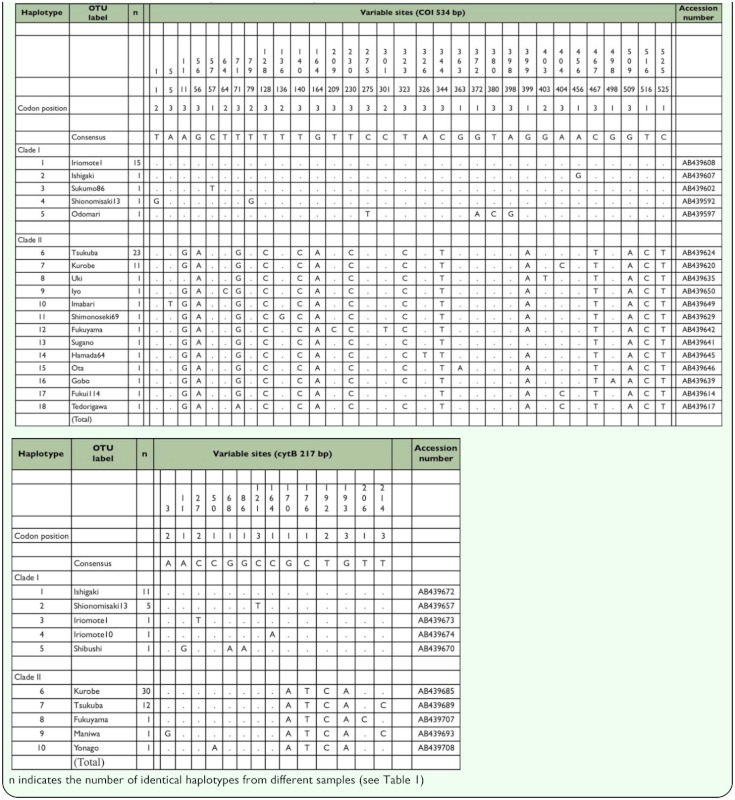
Sequence variation in the *COI* and *cytB* regions of the mitochondrial DNA of *Pilophorus typicus* in Japan. Dots indicate identity with the consensus sequence. Clade names agree with those in Fig. 3.

**Table 3.**
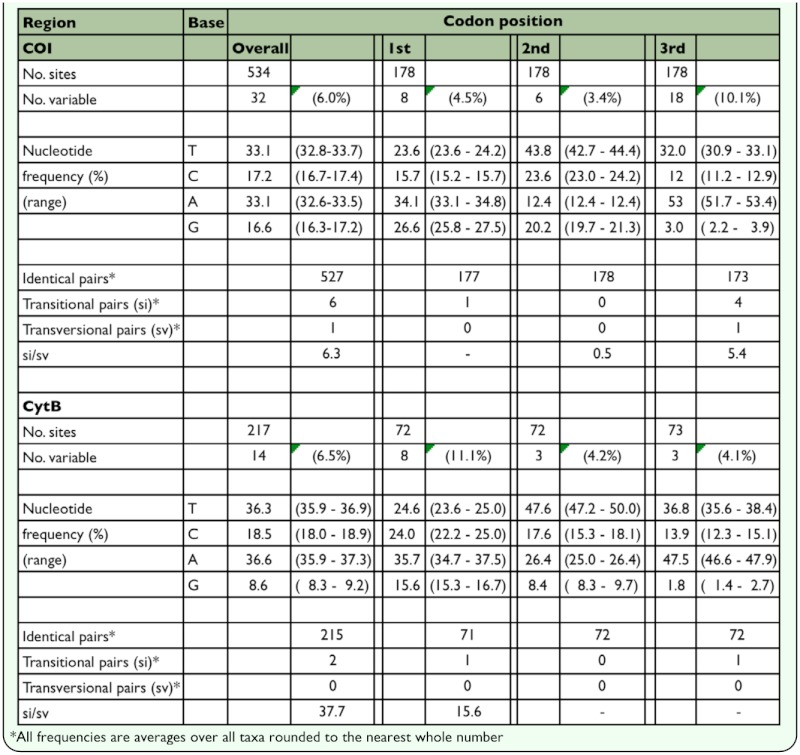
Nucleotide composition in the partial *COI* and *cytB* regions of the mitochondrial DNA of *Pilophorus typicus* in Japan

**Figure 1.**
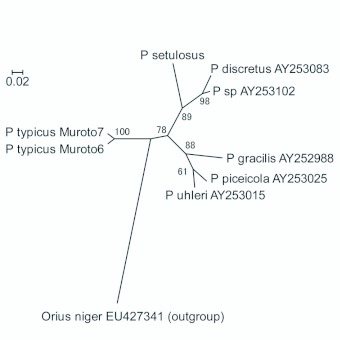
Relationship between Tamura-Nei distances of *COI* and *cytB* regions for each sample. All codon positions are included. Line indicates the set of points on which the distances are equal. High quality figures are available online.

The attributes of nucleotide sequences are summarized in Table 3. The partial *COI* and *cytB* regions exhibited a similar proportion of variable sites (about 6% for each). The most variable codon position was 3rd for the *COI* region and 1st for the *cytB* region. Saturation tests plotting the proportion of different sites against the evolutionary distance showed no clear tendency for saturation at either position of each region (results not shown). As shown in Figure 1, the evolutionary rate appears to be slightly higher in the *COI* region when two sequences from evolutionary distant populations were compared. Within 177 and 72 amino acid residues each translated from the *COI* and *cytB* nucleotide sequences, variability was observed at 10 (5.6%) and 9 (12.5%) sites, respectively. The transition and transversion rate (si/sv) was high (6.3 and 37.7, respectively). The partition homogeneity test on PAUP showed no significant incongruence between the two regions (P = 1.000).

In the preliminary NJ analysis of each region, Modeltest selected the Tamura-Nei model (Tamura and Nei 1993) and the HKY85 model (Hasegawa et al. 1985) for the *COI* and *cytB* regions, respectively. These analyses showed the existence of two distinct clades in the haplotypes of *P. typicus* for each region (results not shown), and the haplotypes composing each clade were identical between the analyses of these regions. Therefore, these regions were assumed to have shared the common evolutionary process and thus all data sets were combined into a single matrix and it was analyzed simultaneously to achieve high resolution of phylogenetic relationships of *P. typicus.*

Combining haplotypes of the two genes, 25 haplotypes were recognized (see Table 1). For combined data of the *COI* and *cytB* regions, Modeltest selected the HKY85 model by the hierarchical likelihood test. Heuristic parameter settings were as follows: empirical base frequencies were A = 0.3300, C = 0.1546, G = 0.1656, and T=0.3498; transition/transversion ratio = 2.2784 (kappa = 5.2384); -ln L (unconstrained) = 1594.45565. The total number of rearrangements tried was 88463, and the score (-ln) of the selected tree was 1705.6616.

**Figure 2.**
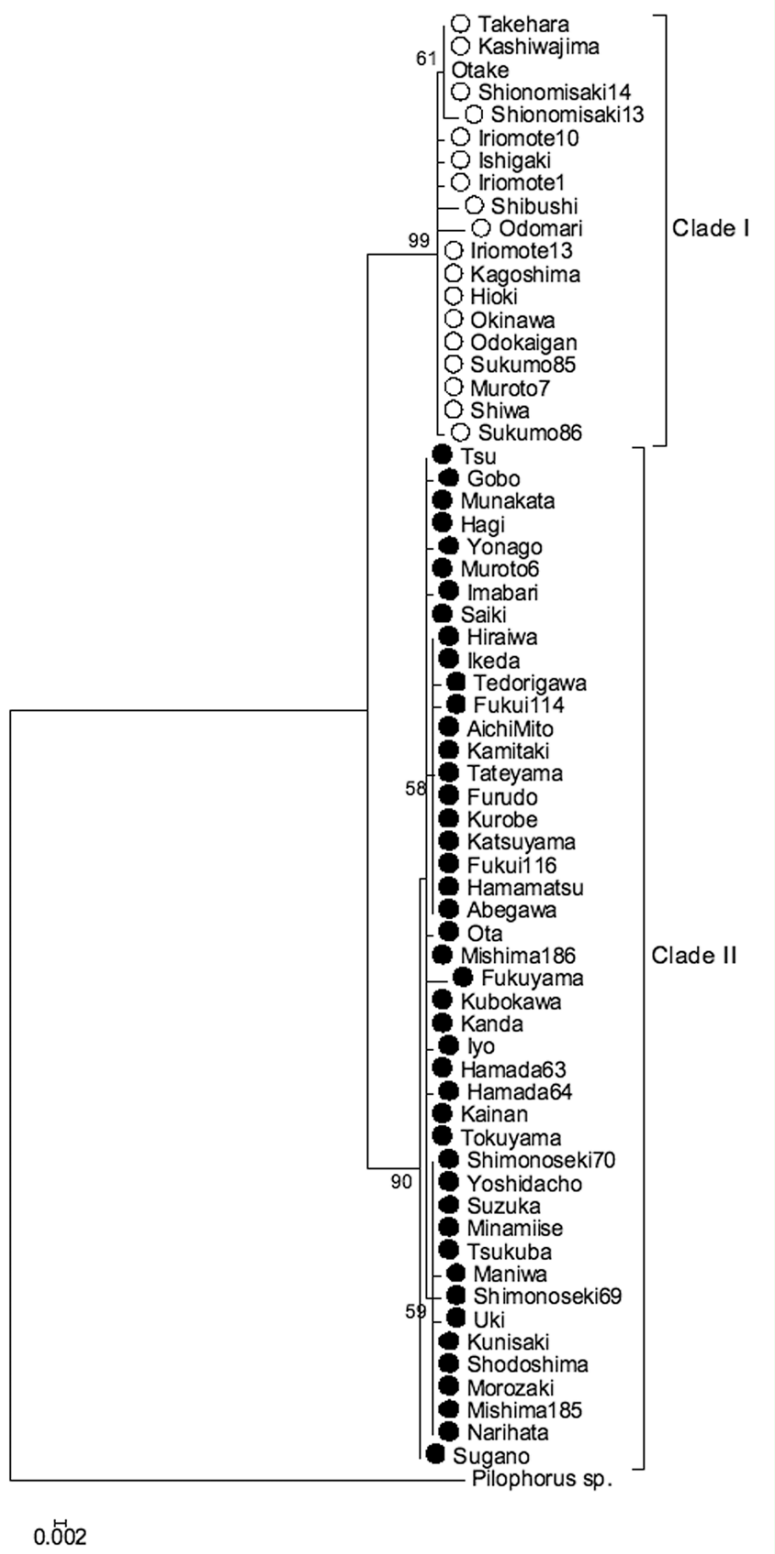
Phylogenetic tree of combined partial sequences of *COI* (534 bp) and *cytB* (217 bp) regions of *Pilophorus typicus* and an outgroup obtained by ML method. Numerals above the branches indicate bootstrap values (>50%, 1000 replicates). Black and white circles correspond with plots in Figure 3. High quality figures are available online.

**Figure 3.**
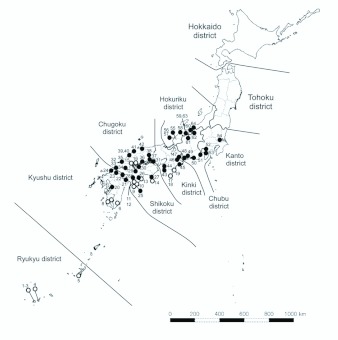
Geographical distribution of the two clades of *COI + Cytb* haplotypes of *Pilophorus typicus* in Japan. Open and closed circles represent Clade I and II, respectively. Numbers correspond to populations in Table 1. High quality figures are available online.

The ML tree showed the existence of two distinct clades in the haplotypes of *P. typicus,* both of which were supported by high (>95%) bootstrap values (Figure 2). The number of base substitutions between the two clades was 1.9% (14 out of 751, Table 2). Within-group genetic diversity was not significantly different between these clades (*COI:* clade I 0.0016 ± 0.0005, II 0.0018 ± 0.0008; *cytB* I 0.0044 ± 0.0022, II 0.0026 ± 0.0019, Tamura-Nei method). Clade I consisted of 19 samples (representing 9 haplotypes) that were found from 14 localities in the southern part of the range of *P. typicus* in Japan, i.e. the Ryukyus and the Pacific coastal parts of Kyushu, Shikoku, and Kinki districts with a few exceptional localities (Otake and Takehara) along the coast of the Seto Inland Sea (Figure 3). On the other hand, Clade II consisted of 45 samples (16 haplotypes) from 41 localities in the northern part of its range: from northern Kyushu to the central part of Honshu through northern Shikoku and most parts of Chugoku and Kinki (Figure 3). Of the 8 localities where 2 or 3 individuals were sampled, 7 localities were represented by either Clades I or II, and one locality in the southern Shikoku (Muroto) included both Clades I and II haplotypes. Considering that both types exist in only a few samples, localities in which both types reside may be more than shown in this result. These results suggest that the two clades have different distribution ranges (Figure 3), but in southwestern parts of Japan individuals of both groups are living sympatrically.

The observed distribution of the two clades suggests discordance between variation in DNA sequences and previously reported morphological variation. A previous study has revealed the existence of two morphologically distinct forms, recognized by a different structure of male genitalia, in *P. typicus* by a broad sampling from East and Southeast Asia including Japan, Taiwan, Malaysia, and Indonesia (Nakatani Y unpublished data; Yamada K, personal communication). However, to date, separation of their distribution ranges has been found only between Ishigaki and Iriomote Islands and no other morphological delimitation within the Japan archipelago. Therefore, it is possible that genitalia structures could change within a short evolutionary period in which mitochondrial DNA sequences scarcely vary.

**Figure 4.**
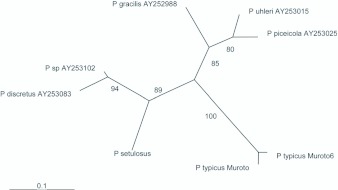
Unrooted tree of partial sequences of *COI* (533 bp) of *Pilophorus typicus* and congeneric species. Numerals above the branches indicate bootstrap values (>50%, 1000 replicates). High quality figures are available online.

Phylogenetic analysis incorporating other species of *Pilophorus* revealed that genetic difference between the two groups was small at the intrageneric level, and thus suggest that they may have been differentiated only recently (Figure 4: GTR+G model; base frequency A = 0.3358, C = 0.1765, G = 0.1630, T = 0.3247; gamma shape parameter = 0.2212; -ln L (unconstrained) = 1594.98329; No. rearrangements = 180; Score of best tree = 1974.17678). Though this phylogenetic proximity does not immediately reflect the degree of reproductive isolation (e.g. Palumbi and Metz 1991), phylogenetically close populations may tend to hybridize more easily than distant ones (cf. Coyne and Orr 1997; Tubaro and Lijtmaer 2002). Therefore, scrutiny of reproductive isolation between the two groups should be investigated to infer the possible risks of disturbing the genetic structures of local populations through genetic introgression. Moreover, introducing genetically different strains may disturb the environment through secondarily damaging nontarget insects (Howarth 1991; Simberloff and Stiling 1996). Hybridization might enhance this process since it serves as a source of new variation. Before introducing *P. typicus* as a biological control agent for crop pests, the details of their ecological aspects such as potential host preference of these two groups and their reproductive compatibility should be adequately investigated, and the genetic and ecological impacts on the agroecosystem of application sites should be assessed.

## Abbreviations

***COI***,: cytochrome oxidase subunit I
**cytB**,: cytochrome B
**ML**,: maximum likelihood
**NJ,**: neighborjoining
**TBR,**: tree-bisection-reconnection

## References

[bibr01] Coyne JA, Orr HA (1997). ‘Patterns of speciation in *Drosophila’* revisited.. *Evolution*.

[bibr02] Distant WL (1909). Descriptions of Oriental Capsidae.. *Annals and Magazine of Natural History*.

[bibr03] Farris JS, Källersjö M, Kluge AG, Bult C (1994). Testing significance of incongruence.. *Cladistics*.

[bibr04] Farris JS, Källersjö M, Kluge AG, Bult C (1995). Constructing a significance test for incongruence.. *Systematic Biology*.

[bibr05] Folmer O, Black M, Hoeh W, Lutz R, Vrijenhoek R (1994). DNA primers for amplification of mitochondrial cytochrome c oxidase subunit I from diverse metazoan invertebrates.. *Molecular Marine Biology and Biotechnology*.

[bibr06] Hasegawa M, Kishino H, Yano T (1985). Dating of the human-ape splitting by a molecular clock of mitochondrial DNA.. *Journal of Molecular Evolution*.

[bibr07] Havill NP, Foottit RG, von Dohlen CD (2007). Evolution of host specialization in the Adelgidae (Insecta: Hemiptera) inferred from molecular phylogenetics.. *Molecular Phylogenetics and Evolution*.

[bibr08] Hebert PDN, Cywinska A, Ball SL, deWaard JR (2003). Biological identifications through DNA barcodes.. *Proceedings of the Royal Society B: Biological Sciences*.

[bibr09] Howarth FG (1991). Environmental impacts of classical biological control.. *Annual Review of Entomology*.

[bibr10] Ito K, Nishikawa H, Shimada T, Ogawa K, Minamiya Y, Hayakawa H, Fukuda T, Arakawa R (2009). Molecular identification of genotypes of *Pilophorus typicus* (Heteroptera: Miridae) in Japan using PCR-RFLP analysis of mitochondrial DNA.. *Environment Control in Biology*.

[bibr11] Muraji M, Kawasaki K, Shimizu T (2000a). Nucleotide sequence variation and phylogenetic utility of the mitochondrial *COI* fragment in anthocorid bugs (Hemiptera: Anthocoridae).. *Applied Entomology and Zoology*.

[bibr12] Muraji M, Kawasaki K, Shimizu T (2000b). Phylogenetic utility of nucleotide sequences of mitochondrial 16S ribosomal RNA and cytochrome b genes in anthocorid bugs (Heteroptera: Anthocordiae).. *Applied Entomology and Zoology*.

[bibr13] Muraji M, Kawasaki K, Shimizu T (2001). Nucleotide sequence variation and use of mitochondrial DNA for phylogenetic analyses in Anthocorid bugs (Hemiptera: Anthocoridae).. *Japan Agricultural Research Quarterly*.

[bibr14] Nei M, Kumar S (2000). *Molecular Evolution and Phylogenetics.*.

[bibr15] Palumbi SR, Metz EC (1991). Strong reproductive isolation between closely related tropical sea urchins (genus *Echinometra*).. *Molecular Biology and Evolution*.

[bibr16] Phillips CB, Vink CJ, Blanchet A, Hoelmer KA (2008). Hosts are more important than destinations: What genetic variation in
*Microctonus aethiopoides* (Hymenoptera: Braconidae) means for foreign exploration for natural enemies.. *Molecular Phylogenetics and Evolution*.

[bibr17] Pons J, Barraclough T, Theodorides K, Cardoso A, Vogler A (2004). Using exon and intron sequences of the gene Mp20 to resolve basal relationships in *Cicindela* (Coleoptera: Cicindelidae).. *Systematic Biology*.

[bibr18] Posada D, Crandall KA (1998). Modeltest: testing the model of DNA substitution.. *Bioinformatics*.

[bibr19] Schuh RT (1984). Revision of the Phylinae (Hemiptera, Miridae) of the Indo-Pacific.. *Bulletin of the American Museum of Natural History*.

[bibr20] Simberloff D, Stiling P (1996). How risky is biological control?. *Ecology*.

[bibr21] Simmons RB, Weller SJ (2001). Utility and evolution of cytochrome b in insects.. *Molecular Phylogenetics and Evolution*.

[bibr22] Smith PT (2005). Mitochondrial DNA variation among populations of the glassywinged sharpshooter, *Homalodisca coagulata.*. *Journal of Insect Science*.

[bibr23] Swofford DL (2003). *PAUP***. Phylogenetic Analysis Using Parsimony (***and Other Methods). Version 4.*.

[bibr24] Tamura K, Dudley J, Nei M, Kumar S (2007). MEGA4: Molecular evolutionary genetics analysis (MEGA) software version 4.0.. *Molecular Biology and Evolution*.

[bibr25] Tamura K, Nei M (1993). Estimation of the number of nucleotide substitutions in the control region of mitochondrial DNA in humans and chimpanzees.. *Molecular Biology and Evolution*.

[bibr26] Thompson JD, Higgins DG, Gibson TJ (1994). CLUSTAL W: improving the sensitivity of progressive multiple sequence alignment through sequence weighting, positions-specific gap penalties and weight matrix choice.. *Nucleic Acids Research*.

[bibr27] Tubaro PL, Lijtmaer DA (2002). Hybridization patterns and the evolution of reproductive isolation in ducks.. *Biological Journal of the Linnean Society*.

[bibr28] Yasunaga T, Yasunaga T, Takai M, Kawasawa T (2001). Genus *Pilophorus* Hahn, 1826.. *A Field Guide to Japanese Bugs II: Terrestrial Heteropterians*.

